# Magnetic mesoporous bioactive glass for synergetic use in bone regeneration, hyperthermia treatment, and controlled drug delivery†

**DOI:** 10.1039/c9ra09349d

**Published:** 2020-06-04

**Authors:** Muhammad Saif Ur Rahman, Muhammad Asif Tahir, Saima Noreen, Muhammad Yasir, Ijaz Ahmad, Muhammad Bilal Khan, Khawajah Waqar Ali, Muhammad Shoaib, Ali Bahadur, Shahid Iqbal

**Affiliations:** Zhejiang University-University of Edinburgh Institute, Zhejiang University Haining People's Republic of China; Clinical Research Center, The Second Affiliated Hospital, Zhejiang University, School of Medicine Hangzhou 310009 Zhejiang Province China; Department of Chemistry, University of Agriculture Faisalabad 38000 Pakistan; Department of Chemistry, University of Lahore Lahore 54770 Pakistan; Department of Chemistry, Government Postgraduate College Samanabad Faisalabad 38000 Pakistan relyables@gmail.com; Allama Iqbal Open University Islamabad Pakistan; Department of Transdisciplinary Studies, Graduate School of Convergence Science and Technology, Seoul National University Seoul 16229 South Korea alibahadur138@snu.ac.kr; School of Chemistry and Materials Engineering, Huizhou University Huizhou 516007 Guangdong China

## Abstract

A combination of chemotherapy with hyperthermia can produce remarkable success in treating advanced cancers. For this purpose, magnetite (Fe_3_O_4_)-doped mesoporous bioactive glass nanoparticles (Fe_3_O_4_-MBG NPs) were synthesized by the sol–gel method. Fe_3_O_4_-MBG NPs were found to possess spherical morphology with a size of approximately 50 ± 10 nm and a uniform pore size of 9 nm. The surface area (309 m^2^ g^−1^) was sufficient for high drug loading capacity and mitomycin C (Mc), an anticancer drug, was entrapped in the Fe_3_O_4_-MBG NPs. A variable rate of drug release was observed at different pH values (6.4, 7.4 & 8.4) of the release media. No significant death of normal human fibroblast (NHFB) cells was observed during *in vitro* analysis and for Mc-Fe_3_O_4_-MBG NPs considerable inhibitory effects on the viability of cancer cells (MG-63) were observed. When Fe_3_O_4_-MBG NPs were immersed in simulated body fluid (SBF), hydroxycarbonate apatite (HCA) was formed, as confirmed by XRD and FTIR spectra. A negligible value of coercivity and zero remanence confirms that Fe_3_O_4_-MBG NPs are superparamagnetic. Fe_3_O_4_-MBG NPs showed a hyperthermia effect in an alternating magnetic field (AMF), and a rise of 11.5 °C in temperature during the first 6 min, making it suitable for hyperthermia applications. Fe_3_O_4_-MBG NPs expressed excellent biocompatibility and low cytotoxicity, therefore, they are a safe biomaterial for bone tissue regeneration, drug delivery, and hyperthermia treatment.

## Abstract 2

A combination of chemotherapy with hyperthermia can produce remarkable success in treating advanced cancers.

## Graphical abstract

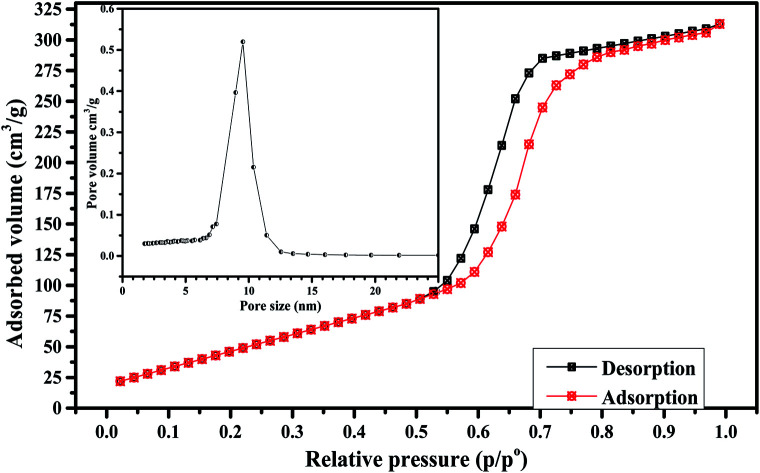

## Introduction

Bone is a self-healing tissue in minor defects but treating larger defects due to trauma, osteoporosis, tumour removal, infection or thinning is still a clinical and socio-economical challenge. Therefore, the need of the hour is a synthetic bone graft that can also overcome the limitations of other bone treatment methods such as autografts and allografts, which suffer from donor site morbidity, weak osteoinductivity, and potential risk of infection.^1–4^ For this purpose, a wide variety of biomaterials were prepared for bone-tissue regeneration and fascinating are those which exhibit multifunctional abilities such as osteoconductivity, osteogenesis, and angiogenesis.

Moreover, sometimes a huge amount of bone is removed due to cancer and it is impossible to harvest such a greater mass of bone; therefore, tissue regeneration is a viable alternative.^5,6^ But even the bone regeneration is substantially hindered by infections that were conventionally treated by antibiotic administration and wound drainage. However, these methods were mostly ineffective and resulted in further complications leading to extra surgeries, which cause pain and economic cost. Subsequently, a multifunctional biomaterial was needed to solve all these problems and would represent a valuable solution in preventing post-surgical infections along with bone regeneration.^7–9^

Therefore nanobioactive glass such as mesoporous bioactive glass (MBG) is one of the most promising biomaterials which possess exceptional osteoinductive behavior and can form a bond with hard and soft tissues through hydroxycarbonate apatite (HCA).^10–12^ It also plays an important role in regenerative medicines, drug carriers, and biosensors. This wide variety of applications of MBG depend upon the surface area, morphology, pore size, stoichiometric ratio, crystallinity, composition, and crystal size distribution. To obtain tailor-made properties, different compositions were studied and doped with different ions. Different metallic (Ag, K, Mg, Sr, Cu, and Co) and non-metallic (B) ions impart several biological functions such as stimulation of osteogenesis, angiogenesis, and anti-bacterial activities.^13–15^

Although MBG is successfully used for bone regeneration and delivery of anti-cancer drugs to the cancerous bones, it does not kill the cancer cells itself. Therefore, in this study, MBG was doped with magnetite (Fe_3_O_4_) for its synergetic use hyperthermia treatment of cancer cells. After implantation in the affected region, it is exposed to the alternating magnetic field and produces heat. Thus, relatively high temperature is maintained (>43 °C) in the region of neoplastic tissue and malignant cells are selectively killed.^16–18^ Thus Fe_3_O_4_-MBG NPs (51SiO_2_·18CaO·20Na_2_O·4P_2_O_5_·7Fe_3_O_4_ mol%) were prepared and used for drug delivery, bone-tissue regeneration, and hyperthermia treatment.

## Materials and methods

### Materials

For the preparation of magnetite doped mesoporous bioglass, ferric chloride (FeCl_3_·6H_2_O, 99%), ferrous chloride (FeCl_2_·4H_2_O, 99%), ammonia solution (NH_3_, 25%), hydrochloric acid (HCl, 37%), tetraethyl orthosilicate (TEOS, 98%), triethyl phosphate (TEP, 99.8%), calcium nitrate tetrahydrate (Ca(NO_3_)_2_·4H_2_O, 99%), sodium carbonate (Na_2_CO_3_, 99.99%), pluronic P-123, and absolute ethanol (C_2_H_5_OH, 99.9%), were purchased from Sigma-Aldrich.

### Preparation of magnetite (Fe_3_O_4_) nanoparticles

10 mL aqueous solution of 1 M FeCl_2_·4H_2_O and 20 mL of 1 M FeCl_3_·6H_2_O (3 mmol) were mixed and stirred under an inert atmosphere of nitrogen. When 100 mL of ammonia solution (1 M) was added to it, dark brown precipitates were formed which were further stirred for 4 h. After washing with water and 0.05 N HCl precipitates were dried at room temperature to obtain the magnetite (Fe_3_O_4_) nanoparticles.

### Preparation of magnetite doped mesoporous bioactive glass (Fe_3_O_4_-MBG)

For preparing Fe_3_O_4_-MBG (51SiO_2_·18CaO·20Na_2_O·4P_2_O_5_·7Fe_3_O_4_ mol%), TEOS was added to a mixture of 5 g of P123, 10 mL of 0.2 N HNO_3_, 50 mL of absolute ethanol and 500 mL of deionized water. The mixture was stirred for 2 h in an inert atmosphere then TEP, Ca(NO_3_)_2_·4H_2_O, and Na_2_CO_3_ were added and stirred with a time interval of 45 min for each component. Then already prepared Fe_3_O_4_ NPs were added, and the mixture was stirred for 1 h so that sol is formed. When ammonia solution (25%) was added dropwise a thick gel was formed, which was further stirred for 2 h, aged overnight at room temperature, dried at 100 °C in a vacuum oven and finally calcined at 350 °C for 4 h to get Fe_3_O_4_-MBG.^19^

### Characterizations

FTIR spectrum was taken by a Nicolet iS10 FTIR spectrometer and XRD analysis was performed by using an X-ray diffractometer (PANalytical, X'Pert Pro, Almelo, Netherlands); having a Cu Kα radiation source operated at 40 kV. The surface area and pore size of Fe_3_O_4_-MBG NPs were measured by using the BJH (ASAP 2010) and BET (Micromeritics Instrument Corp, Gemini V2.0 analyses). Particle size, shape, morphology, and elemental analysis were achieved by using SEM, EDX (Hitachi S3400N), and TEM (JEM-1400 Plus). UV/Vis spectrophotometer (Shimadzu UV-265) was used to determine the concentration of the drug. Zeta potential and the surface charge was determined with the help of Zetasizer ZS (Malvern Instruments, Malvern, UK). The magnetic properties were measured by using the Quantum Design PPMS magnetometer.

### Drug loading and release study

For drug loading, one gram of Fe_3_O_4_-MBG was stirred with 500 mg of mitomycin C (Mc) in 100 mL of deionized water for 4 h. Solid was filtered and dried at room temperature to obtain Mc-loaded Fe_3_O_4_-MBG (Mc-Fe_3_O_4_-MBG) NPs. For the mitomycin C release studies, 500 mg of Mc-Fe_3_O_4_-MBG was placed in a glass vial having 5 mL of SBF. After a specific time-interval, 1 mL of this solution was taken out, filtered and concentration of drug was measured with the help of UV/Vis spectrophotometer. The same volume of fresh SBF was also added in the vial to keep the volume of the solution constant.

### Bone tissue regeneration study

For the investigation of bone-forming ability, 500 mg of the Fe_3_O_4_-MBG NPs were immersed in SBF and incubated at 37 °C. After one week, Fe_3_O_4_-MBG was filtered, washed gently with acetone, and dried at room temperature.^20^ The samples were subjected to XRD and FTIR analysis to explore the HCA formation.

### MTT assay

For cytotoxicity evaluation of Fe_3_O_4_-MBG NPs, different concentrations were applied against normal human fibroblast (NHFB) cell line and cell viability was determined by MTT assay. Moreover, their inhibitory effects on the viability of the osteosarcoma cell line (MG-63) were also determined along with mitomycin C drug, and Mc-Fe_3_O_4_-MBG NPs. For this purpose, 10^4^ cells were seeded per well in 96 well plate and after 24 h these cells were exposed with different concentrations of Mc, Fe_3_O_4_-MBG NPs, and Mc-Fe_3_O_4_-MBG NPs for 72 hours. After subsequent incubation 0.5 mg mL^−1^ of 100 μL of MTT was added in each well. The absorbance of the resultant formazan product was determined by using the microplate reader (Spectra MAX, USA) at 490 nm and percent viability was measured by Graph Pad Prism 6.0 (GraphPad, San Diego, CA USA).

### Alkaline phosphatase activity and osteocalcin assay

For ALP measurement osteoblast cells were cultured and treated with Fe_3_O_4_-MBG, for 48, 72, and 120 h. The cells were treated according to the previously described protocol and cell lysate was used for the ALP activity, according to the manufacturer kit (ab83369). A standard curve was calculated using *p*-nitrophenol and ALP activity unit was calculated.

For osteogenic assay cells were cultured on Fe_3_O_4_-MBG in DMEM without FBS in 6 well plates. After 120 h, culture medium from each well was aspirated and assayed by following a Human Osteocalcin ELISA kit (Biomedical Technologies Inc, Tyne & Wear, UK).^21^

### Hyperthermia study

The hyperthermia property of Fe_3_O_4_-MBG NPs was studied by using AC applicator DM100 by nB nanoscale Biomagnetics, working at the frequency of 220–260 kHz and magnetic field amplitude (*H*_o_) up to 23.9 kAm^−1^ (300 guess). 1 mg mL^−1^ of Fe_3_O_4_-MBG was suspended in deionized water at room temperature in a glass tube. The alternating magnetic field of frequency 250 kHz and magnetic field strength at 6 kAm^−1^ were applied to the Fe_3_O_4_-MBG solution for 20 min adiabatically by keeping the surrounding temperature at 25 °C. The temperature was measured using a fiber optic temperature probe. The specific absorption rate (SAR) was measured by the following equation.1
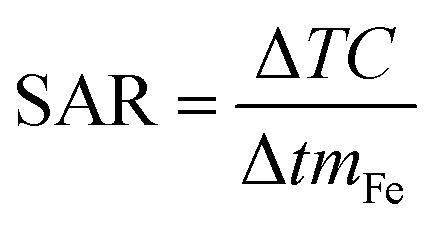


In this equation, *C* (4.18 J g^−1^ °C^−1^) represent specific heat of water, Δ*T* is temperature change, *t* is the change in time, *m*_Fe_ is the fractional mass of Fe in Fe_3_O_4_-MBG.

### Statistical analysis

Statistical analysis performed by using Graphpad Prism. *P* < 0.05, was regarded as significant and data is expressed as mean ± SE.

## Results and discussion

**Fig. 1:**
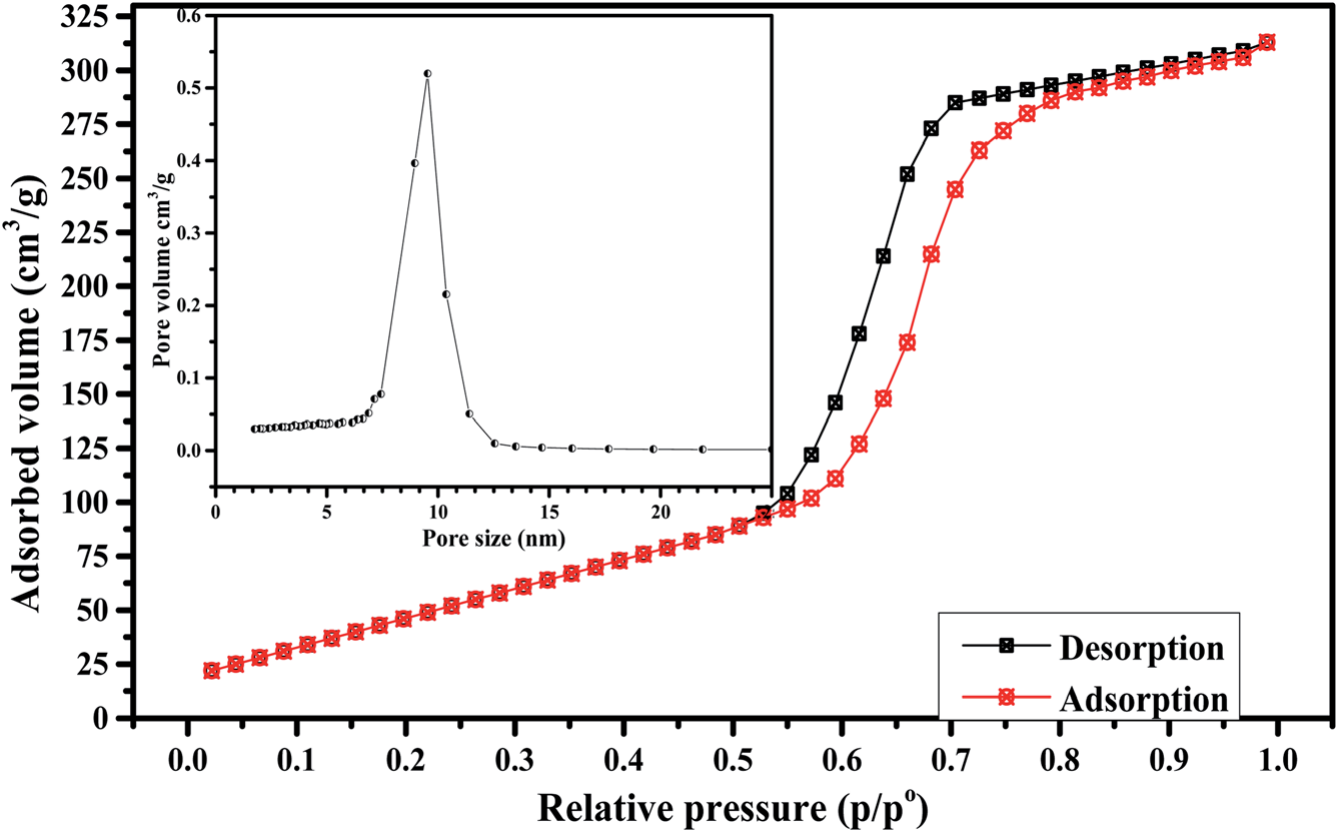
BET surface area measurements (inset) pore size distribution of Fe_3_O_4_-MBG.

**Fig. 2:**
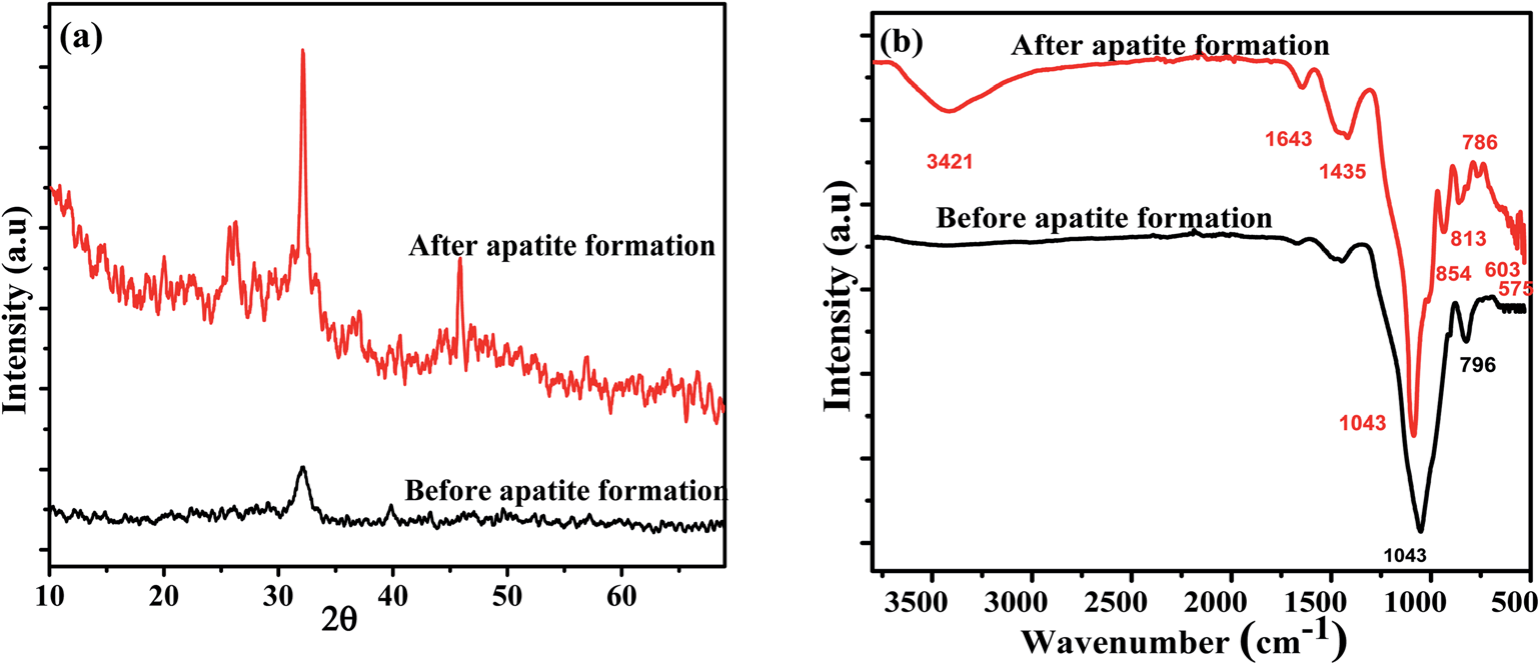
(a) XRD analysis (b) FTIR spectra of Fe_3_O_4_-MBG before and after apatite formation.

The prepared Fe_3_O_4_-MBG NPs were subjected to various characterizations and screening before using them as a drug delivery carrier. The results of BET nitrogen adsorption–desorption analysis for the Fe_3_O_4_-MBG are given in Fig. 1 as type of IVa isotherm, confirming the mesoporous structure. The BJH analysis confirms the narrow pore size distribution at 9.52 nm and BET analysis shows that the surface area is 309 m^2^ g^−1^. The XRD analysis was performed to evaluate any crystallinity in the Fe_3_O_4_-MBG NPs. Before immersion in SBF diffractogram shows one broad diffraction halo which confirms its amorphous nature. The apatite formation *i.e. in vitro* test for bone formation was confirmed by immersing the Fe_3_O_4_-MBG NPs in SBF and X-ray diffractogram is presented in Fig. 2(a). These peaks were matched with the JCPDS standard for HA 9-432 and HCA 9-272 which confirms the apatite formation. The intensity and sharpness of the peaks at a 2*θ* value of 26° (002), 32° (211), and 46° (222) represent the bone-forming ability of Fe_3_O_4_-MBG, and therefore, it can be used in bone regeneration and repair.^22,23^ Immersion of Fe_3_O_4_-MBG in SBF is useful *in vitro* test for the confirmation of apatite-forming ability. FTIR spectra of Fe_3_O_4_-MBG before and after mineralization is shown in Fig. 2(b). The peaks at 1043, 796 cm^−1^ are associated with the Si–O vibration. After immersion in SBF for one week, the vibrational bands of carbonated hydroxyapatite were also observed. The vibrational peaks at 1435 and 854 cm^−1^ are assigned to C–O vibration bands of carbonate. The twin bands at 603 and 575 cm^−1^ are assigned to P–O vibration bands of a phosphate group, at 1643 and 3421 are assigned to the O–H group.^24,25^

**Fig. 3:**
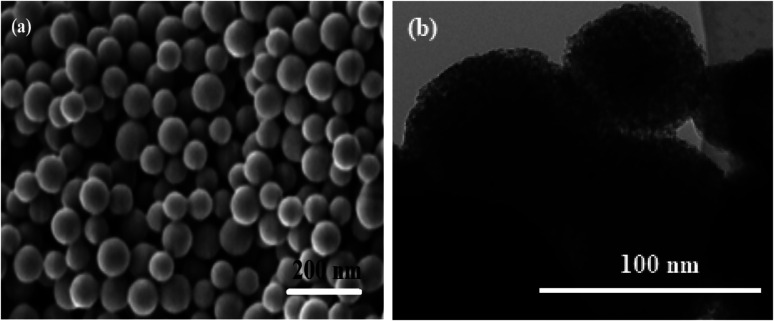
(a) SEM image and (b) TEM image.

The morphology of the Fe_3_O_4_-MBG was analyzed and the SEM and TEM images are shown in Fig. 3, exhibiting spherical and uniform morphology with a particle size around 50 nm. Mitomycin C (Mc) anticancer drug was loaded to the Fe_3_O_4_-MBG NPs and the anti-cancer effect of Mc-Fe_3_O_4_-MBG NPs with different concentrations was evaluated against MG-63 cells.

**Fig. 4:**
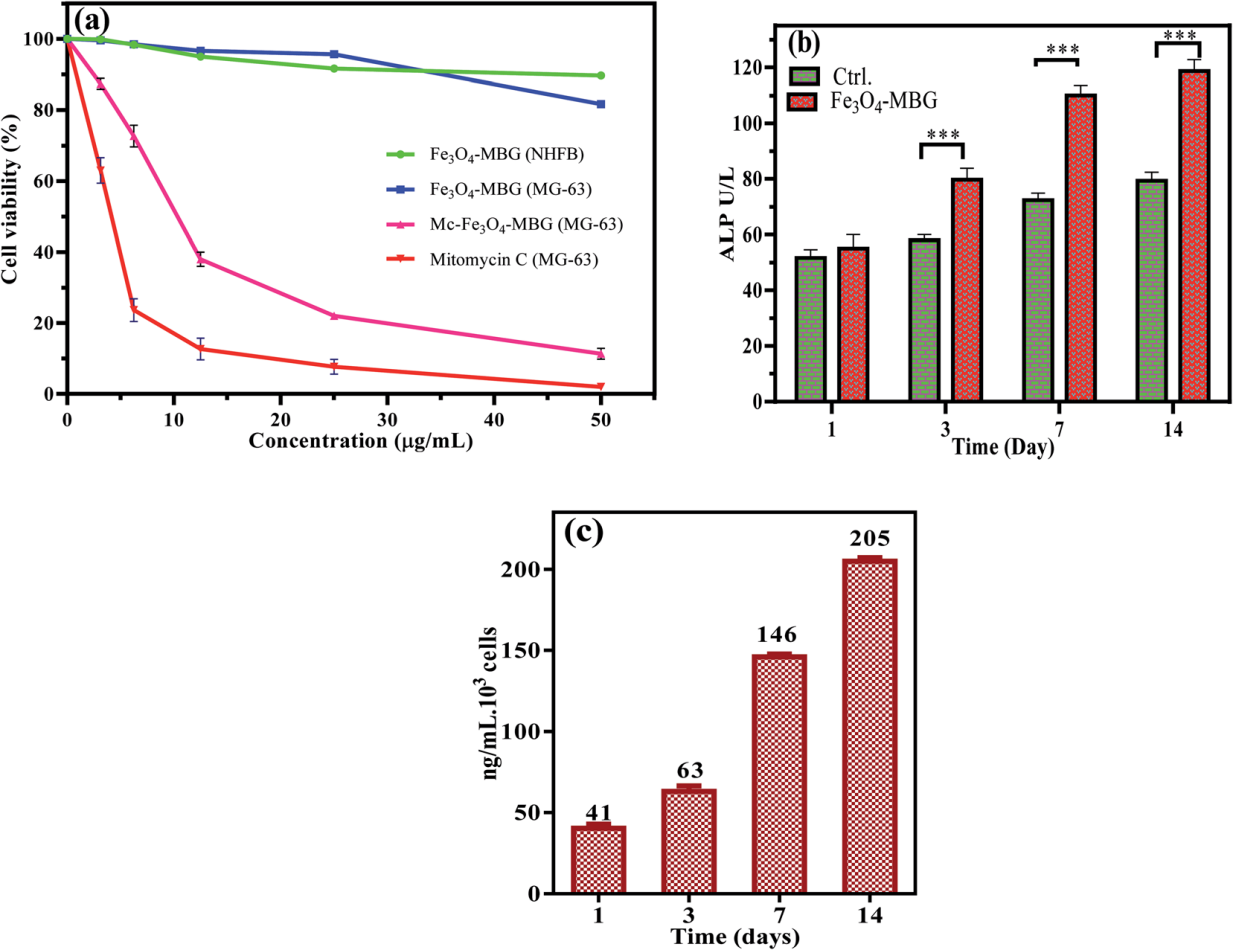
(a) MTT assay of Fe_3_O_4_-MBG against NHFB and MG-63 cells, Mc-Fe_3_O_4_-MBG against MG-63 cancer cells (b) ALP activity and (c) osteocalcin level reflects the bone-forming property of Fe_3_O_4_-MBG.

MTT results revealed that the Mc-Fe_3_O_4_-MBG posed a substantial inhibitory effect on the viability of osteosarcoma cells. The inhibitory effect on the viability of osteosarcoma cells also depends upon the concentration of Mc-Fe_3_O_4_-MBG NPs. MG-63 viability in response to different dose concentrations indicates that Fe_3_O_4_-MBG alone has insignificant toxicity while Mc-Fe_3_O_4_-MBG NPs have very low IC_50_ value (12.19 μg mL^−1^) as shown in Fig. 4(b). It suggests that Mc-Fe_3_O_4_-MBG has significant antiproliferative effects on MG-63 cells. The effects of Fe_3_O_4_-MBG on normal human fibroblast (NHFB) cell lines were also examined by MTT assay and no significant effect on cell proliferation was observed at any concentration which suggests that it is non-toxic, biocompatible, and is safer even at higher concentration proposing to be used for biomedical applications and drug delivery.^26,27^

It is interpreted that surface area plays an important role in the release of ions and apatite formation. As the surface area of Fe_3_O_4_-MBG is relatively higher, therefore the rate of dissolution is also higher. Fe_3_O_4_-MBG is stable in SBF solution, did not dissolve in SBF but Mitomycin C (Mc) anticancer drug which is loaded into Fe_3_O_4_-MBG NPs show dissolution in SBF. The high dissolution rate of the drug is due to the high surface area and porous nature of Fe_3_O_4_-MBG NPs which facilitate the solvent penetration into the matrix of Fe_3_O_4_-MBG.

Biochemical analysis reveals that osteogenic ability and osteoblast differentiation was investigated by ALP activity, which indicates the onset and initial differentiation of osteoblast cells. Over time, this activity is diminished showing the onset of mineralization which occurs in later stages of osteoblast differentiation. ALP activity works by modulating the phosphate metabolism during the bone-forming process. ALP activity of cultured osteoblast cells on Fe_3_O_4_-MBG is considerably higher at day 1 and 3 but after that, the increase in value is very less on the 7^th^ and 14^th^ day which advocates the process of bone mineralization.^28^ To interpret the mature osteoblast phenotype formation, ALP activity alone is less useful, therefore the level of OC is also taken into consideration.^29–31^

Osteocalcin (OC) is synthesized in the bone by the osteoblasts and its level reflects the rate of bone formation. Precisely it is an indicator of the later stage of osteoblastic activity and illustrates the mature lineage of osteocytes.^32^ OC level results in osteoblast differentiation and the osteocytes actively produce mineralized bone tissue. As shown in ESI Fig. S1,† the value of OC is considerably higher on the 7^th^ day and 14^th^ day and this higher level of OC in response to Fe_3_O_4_-MBG suggest its characteristic bone-forming ability and potential to be used as the material for bone repairing and regeneration.^33^

**Fig. 5:**
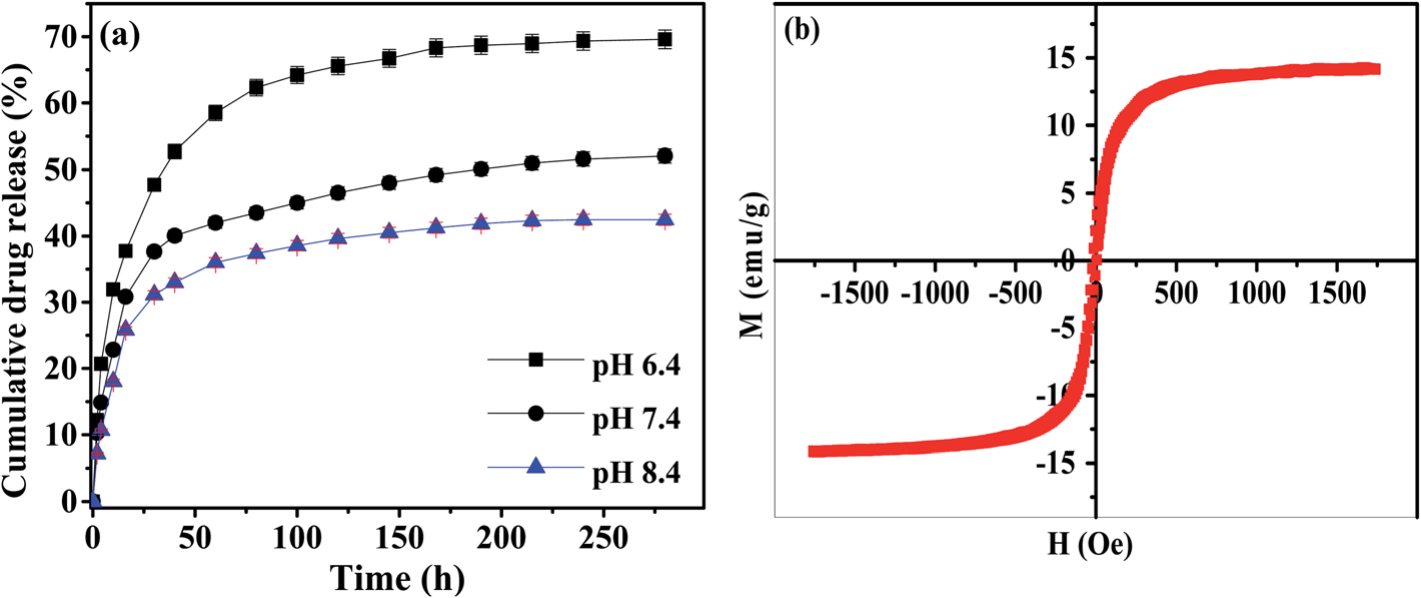
(a) Cumulative drug release (%) as a function of time for Fe_3_O_4_-MBG (b) *M*–*H* loop for Fe_3_O_4_-MBG at room temperature.

The Fe_3_O_4_-MBG shows an admirable loading efficiency of 93% for mitomycin C, which is quite higher as compared to some previous carriers. Most of the drug was loaded into the inner pores and some are adsorbed on the outer surface. The drug can also form complex with that of metal ions present in the composition.^34,35^ As 93% of drug loading efficiency is very high, therefore it was expected that the drug release will also be higher. The maximum cumulative release of 69.6% was observed for the pH of 6.4 and the lowest release of 42% was observed at a pH of 8.4 as shown in Fig. 5(a). It is hypothesized that there is a strong interaction of the drug with that of glass particles, therefore, the overall release of the drug is lower. Its release rate can be adjusted by changing the pH values. Advantage can be taken from the lower pH of cancer affected body parts and drug is specifically released there. This system is an effective drug carrier for curing of tumors and drug release to those parts of the body which are affected by cancer. Maximum release against different pH values is in order 6.4 > 7.4 > 8.4. As the drug delivery is affected by the pH of the release media, therefore, the system can be called as a pH-responsive system.^36–39^ The magnetic study like coercivity (*H*_c_) and saturation magnetization (*M*_s_) of Fe_3_O_4_-MBG were measured by vibrating sample magnetometer (VSM) with the magnetic field of ±5 kOe at room temperature. *M*–*H* loops of Fe_3_O_4_-MBG clearly showed that the Fe_3_O_4_-MBG is superparamagnetic with a negligible value of coercivity and zero remanences suitable for hyperthermia as shown in Fig. 5(b). Saturation magnetization value is 0.087 emu mg^−1^ of Fe (Fe is 0.16 mg mg^−1^ of Fe_3_O_4_-MBG) which is smaller than that bulk value of Fe (0.092 emu mg^−1^). It was due to the spin-glass-like phase (canting spin) on the surface of Fe_3_O_4_-MBG because of oxygen vacancies and lower coordination numbers at the surface.

**Fig. 6:**
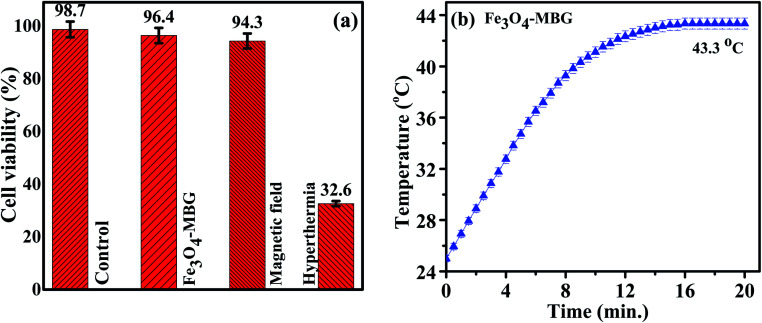
(a) MTT assay (b) temperature kinetics of magnetic hyperthermia study of Fe_3_O_4_-MBG at *f* = 250 kHz.

For explaining the surface charges of these NPs, the zeta potential was measured, and the value was found to be −18.3 ± 0.44 mV. This value if helpful as it causes repulsion and long-term stability.^40–42^ The hyperthermia graph is plotted between time and temperature as shown in Fig. 6(b). Magnetic study shows that Fe_3_O_4_-MBG is superparamagnetic which generates heat due to Brownian and Neel's spin relaxations under the influence of the alternating magnetic field. Fig. 6(b) shows the relationship between time and temperature of the Fe_3_O_4_-MBG solution. After keeping the Fe_3_O_4_-MBG solution in an alternating magnetic field (AMF) for 20 min, the temperature rises from 25 to 43.3 °C due to magnetic relaxation loss. Fe_3_O_4_-MBG shows a high heating effect, rise 11.5 °C temperature in the first 6 min which makes it suitable for hyperthermia application. The SAR value of Fe_3_O_4_-MBG is 305.45 W g^−1^ (Table 1). The high value of SAR, even at a low concentration of magnetite suggests that hyperthermia temperature (>41 °C) can easily be achieved within 3 min.^17,18,40^ MG-63 cancer cells were kept in an alternating magnetic field along with Fe_3_O_4_-MGB (25 μg mL^−1^) and the cultured dish was fitted in a large size alternating magnetic field coil with a frequency of 250 kHz for 20 min and cytotoxicity was evaluated by MTT assay. In three separate dishes, these cells were subjected to the control conditions, magnetic field, and Fe_3_O_4_-MGB (25 μg mL^−1^). MTT assay results in Fig. 6(a) show that most cancer cells were dead at hyperthermia condition whereas, in the absence of AMF, normal cells show high cell viability. These results show that Fe_3_O_4_-MBG is excellent biocompatibility, high hyperthermia temperature, and low cytotoxicity which make it highly effective heat controlled magnetic hyperthermia for cancer treatment.^41,42^

**Table 1:** Properties of Fe_3_O_4_-MBG

Sample	Size (nm)	Pore size (nm)	Surface area (m^2^ g^−1^)	*M*_s_ (emu g^−1^)	*H*_c_	SAR (W g^−1^)	IC_50_ of Mc-Fe_3_O_4_-MBG (μg mL^−1^)	Loaded drug	Released drug
Fe_3_O_4_-MBG	50 ± 5	9.52	309	14.16	0	305.45	12.19	93%	42–72%

## Conclusions

In this study, a multifunctional magnetic mesoporous bioactive glass was prepared for hyperthermia and controlled drug release of anti-cancer drugs. Magnetite NPs and mesoporous bioactive glass were synthesized to produce (Fe_3_O_4_-MBG) nanoparticles (NPs) of spherical morphology, a uniform pore size of 9 nm, and a surface area of 309 m^2^ g^−1^. Mitomycin C was loaded to the Fe_3_O_4_-MBG which showed different rate of drug release at different pH values (6.4, 7.4, and 8.4) of the release media. The as-synthesized Fe_3_O_4_-MBG showed no significant cytotoxicity when subjected to MTT assay by using the NHFB cell line. After drug loading, considerable inhibitory effects on the viability of the cancer cells (MG-63) were observed with IC_50_ of 12.19 μg mL^−1^. Upon immersion in SBF, hydroxycarbonate apatite exhibited the osteogenic ability as supported by XRD and FTIR spectra. Fe_3_O_4_-MBG showed a negligible value of coercivity and zero remanence which confirmed it to be superparamagnetic in behavior. Fe_3_O_4_-MBG showed the heating effect in AMF and rise 11.5 °C in temperature in the first 6 min makes it suitable for hyperthermia application. All the results demonstrated that Fe_3_O_4_-MBG is biocompatible and nontoxic biomaterial which can be used for bone tissue regeneration, targeted drug delivery in chemotherapy, and hyperthermia treatment.

## Conflicts of interest

There is no conflict of interest to be reported.

## Supplementary Material

RA-010-C9RA09349D-s001
