# Correction: Magnetic mesoporous bioactive glass for synergetic use in bone regeneration, hyperthermia treatment, and controlled drug delivery

**DOI:** 10.1039/d0ra90069a

**Published:** 2020-06-25

**Authors:** Muhammad Saif Ur Rahman, Muhammad Asif Tahir, Saima Noreen, Muhammad Yasir, Ijaz Ahmad, Muhammad Bilal Khan, Khawaja Waqar Ali, Muhammad Shoaib, Ali Bahadur, Shahid Iqbal

**Affiliations:** Zhejiang University-University of Edinburgh Institute, Zhejiang University Haining People’s Republic of China; Clinical Research Center, The Second Affiliated Hospital, Zhejiang University, School of Medicine Hangzhou 310009 Zhejiang Province China; Department of Chemistry, University of Agriculture Faisalabad 38000 Pakistan; Department of Chemistry, University of Lahore Lahore 54770 Pakistan; Department of Chemistry, Government Postgraduate College Samanabad Faisalabad 38000 Pakistan relyables@gmail.com; Allama Iqbal Open University Islamabad Pakistan; Department of Transdisciplinary Studies, Graduate School of Convergence Science and Technology, Seoul National University Seoul 16229 South Korea alibahadur138@snu.ac.kr; School of Chemistry and Materials Engineering, Huizhou University Huizhou 516007 Guangdong China

## Abstract

Correction for ‘Magnetic mesoporous bioactive glass for synergetic use in bone regeneration, hyperthermia treatment, and controlled drug delivery’ by Muhammad Saif Ur Rahman *et al.*, *RSC Adv.*, 2020, **10**, 21413–21419, DOI: 10.1039/C9RA09349D.

The authors regret that the name of one of the authors (Khawaja Waqar Ali) was shown incorrectly in the original article. The corrected author list is as shown above.

The Royal Society of Chemistry apologises for these errors and any consequent inconvenience to authors and readers.

## Supplementary Material

