# Supplementary material for: Magnetic mesoporous bioactive glass for synergetic use in bone regeneration, hyperthermia treatment, and controlled drug delivery
Source: RSC Adv. 2020 Jun 4;10(36):21413–9. doi: 10.1039/c9ra09349d (PMC9054387; doi:10.1039/c9ra09349d)
Supplement: RA-010-C9RA09349D-s001 [file RA-010-C9RA09349D-s001.pdf]

Electronic Supplementary Material (ESI) for RSC Advances.
This journal is © The Royal Society of Chemistry 2020

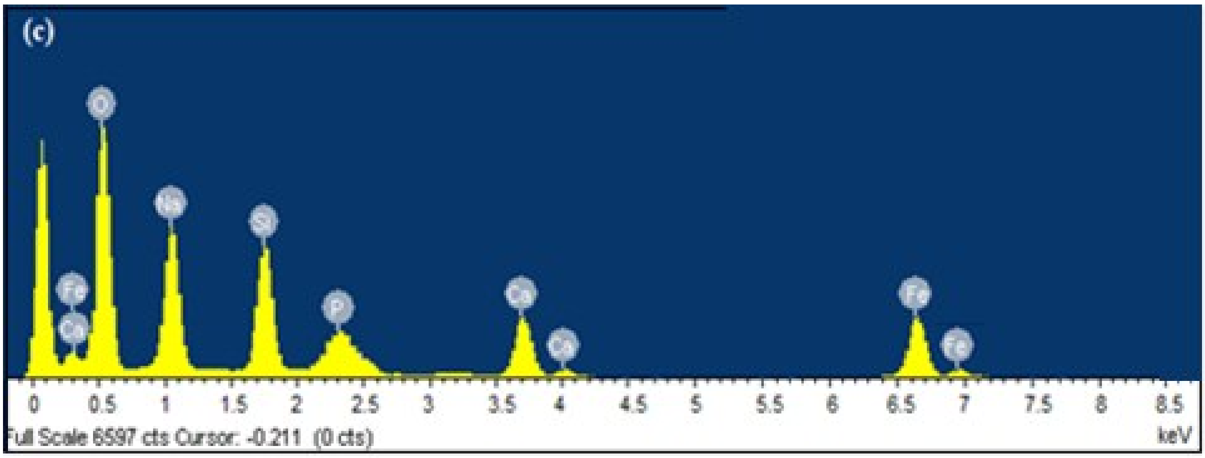

Figure S1: EDX spectra of Fe_3O_4-MBG

Table S1: % composition of Fe_3O_4-MBG

Element	Mole ratio	mass ratio	Wt % based on chemical formula	Wt % based on EDX
Si	51	1428	19	19.3
Ca	18	720	9.6	9.4
Na	40	920	12.2	11.9
P	8	248	3.3	3.1
**Fe**	**21**	**1176**	**15.6**	**15.3**
O	188	3008	40.1	41

Table S2: For Reviewer only

Concentration µg/mL	Cell Viability (%)											
	Fe3O4-MBG (NHFB)			Fe_3O_4-MBG (MG-63)			Mc-Fe_3O_4-MBG (MG-63)			Mitomycin C (MG-63)		
0.00	100	100	100	100	100	100	100	100	100	100	100	100
3.125	100	100	99	99	100	99	89	87	86	67	60	62
6.25	99	98	98	98	98	99	70	72	76	20	25	26
12.50	95	94	96	97	98	95	40	38	36	10	12	16
25.00	92	91	92	96	95	96	21	22	23	7	10	6
50.00	89	90	90	80	82	83	10	11	13	3	2	1
